# Supplementary material for: Causal Relationship between Aging and Anorexia Nervosa: A White-Matter-Microstructure-Mediated Mendelian Randomization Analysis
Source: Biomedicines. 2024 Aug 16;12(8):1874. doi: 10.3390/biomedicines12081874 (PMC11351342; doi:10.3390/biomedicines12081874)
Supplement: Supplementary file 1 [file biomedicines-12-01874-s001.zip › Supplementary Figure/Figure S4/Singe SNP plot of MR analysis for TL to ubm-b-1774.pdf]

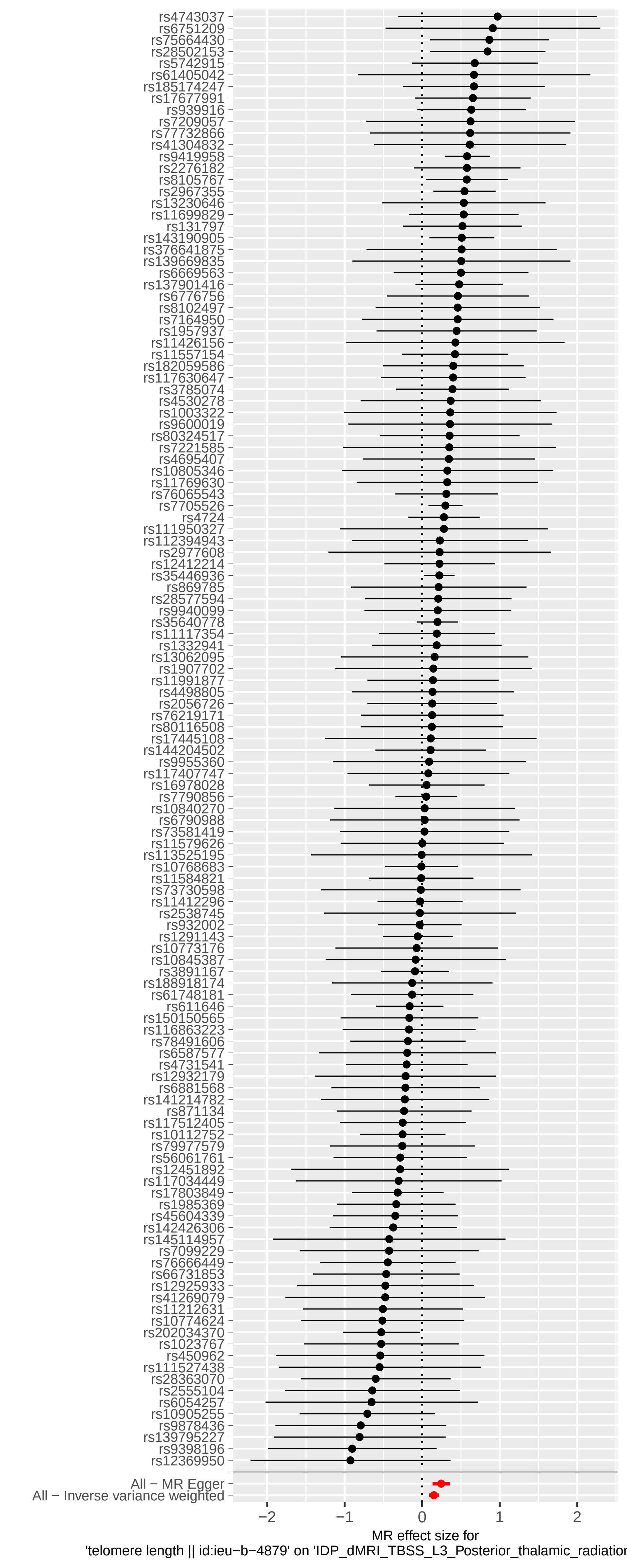
rs4743037
rs6751209
rs75664430
rs28502153
rs5742915
rs61405042
rs185174247
rs17677991
rs939916
rs7209057
rs77732866
rs41304832
rs9419958
rs2276182
rs8105767
rs2967355
rs13230646
rs11699829
rs131797
rs143190905
rs376641875
rs139669835
rs6669563
rs137901416
rs6776756
rs8102497
rs7164950
rs1957937
rs11426156
rs11557154
rs182059586
rs117630647
rs3785074
rs4530278
rs1003322
rs9600019
rs80324517
rs7221585
rs4695407
rs10805346
rs11769630
rs76065543
rs7705526
rs4724
rs111950327
rs112394943
rs2977608
rs12412214
rs35446936
rs869785
rs28577594
rs9940099
rs35640778
rs11117354
rs1332941
rs13062095
rs1907702
rs11991877
rs4498805
rs2056726
rs76219171
rs80116508
rs17445108
rs144204502
rs9955360
rs117407747
rs16978028
rs7790856
rs10840270
rs6790988
rs73581419
rs11579626
rs113525195
rs10768683
rs11584821
rs73730598
rs11412296
rs2538745
rs932002
rs1291143
rs10773176
rs10845387
rs3891167
rs188918174
rs61748181
rs611646
rs150150565
rs116863223
rs78491606
rs6587577
rs4731541
rs12932179
rs6881568
rs141214782
rs871134
rs117512405
rs10112752
rs79977579
rs56061761
rs12451892
rs117034449
rs17803849
rs1985369
rs45604339
rs142426306
rs145114957
rs7099229
rs76666449
rs66731853
rs12925933
rs41269079
rs11212631
rs10774624
rs202034370
rs1023767
rs450962
rs111527438
rs28363070
rs2555104
rs6054257
rs10905255
rs9878436
rs139795227
rs9398196
rs12369950
All − MR Egger
All − Inverse variance weighted
−2
−1
0
1
2
MR effect size for
'telomere length || id:ieu−b−4879' on 'IDP_dMRI_TBSS_L3_Posterior_thalamic_radiatior
